# Book review of “The Biomedical Engineering Handbook” fourth edition, edited by Joseph D. Bronzino, Donald R. Peterson

**DOI:** 10.1186/s12938-015-0119-0

**Published:** 2016-01-06

**Authors:** Robert Koprowski

**Affiliations:** Department of Biomedical Computer Systems, Faculty of Computer Science and Materials Science, Institute of Computer Science, University of Silesia, ul. Będzińska 39, 41-200 Sosnowiec, Poland

**Keywords:** Biomechanics, Biomaterials, Biomedical sensors, Biomedical devices, Biomedical technologies, Image processing, Medical informatics, Molecular biology, Artificial organs

## Abstract

This article is a review of the book “The biomedical engineering handbook”, fourth edition: four volume set (ISBN 9781439825334, 254GBP, 5430 pages) edited by Joseph D. Bronzino and Donald R. Peterson published by the CRC Press Taylor & Francis group in 2015. The content of the book and its importance for biomedical engineering have been discussed in this invited review.

## Book details

Title: “The biomedical engineering handbook”
ISBN: 9781439825334,

Volume I

Print ISBN: 978-1-4398-2530-3
eBook ISBN: 978-1-4398-2531-0

Volume II

Print ISBN: 978-1-4398-2527-3
eBook ISBN: 978-1-4398-2528-0

Volume III

Print ISBN: 978-1-4398-2525-9
eBook ISBN: 978-1-4398-2526-6

Volume IV

Print ISBN: 978-1-4398-2518-1
eBook ISBN: 978-1-4398-2519-8

Price: 254GBP,
Number of pages: 5430,
Number of volumes: 4,
Edited by: Joseph D. Bronzino and Donald R. Peterson,
Published by: CRC Press Taylor & Francis group in 2015.

### Book review

The book submitted for review entitled “The biomedical engineering handbook” was edited by Joseph D. Bronzino and Donald R. Peterson. The book consists of four hardcover volumes amounting in total to 5430 pages. This is another fourth edition. The four volumes present issues relating to the four areas of biomedical engineering: biomedical engineering fundamentals, medical devices and human engineering, biomedical signals, imaging, and informatics, molecular, cellular and tissue engineering. These volumes are divided into chapters, i.e.:

- The first volume (biomedical engineering fundamentals) has 5 chapters comprising 58 articles and the discussed issues concern the following areas: physiologic systems, biomechanics, bioelectric phenomena, neuroengineering,
- The second volume (medical devices and human engineering) has 5 chapters comprising 57 articles and the discussed issues concern the following areas: biomedical sensors, medical instruments and devices, human performance engineering, rehabilitation engineering, clinical engineering,
- The third volume (biomedical signals, imaging, and informatics) has 4 chapters comprising 66 articles and the discussed issues concern the following areas: biosignal processing, medical imaging, infrared imaging, medical informatics,
- The fourth volume (molecular, cellular and tissue engineering) has 9 chapters comprising 93 articles, the discussed issues concern the following areas: molecular biology, transport phenomena and biomimetic systems, physiological modelling, simulation and control, stem cell engineering: an introduction, tissue engineering, artificial organs, drug design, delivery systems, and devices, personalized medicine, ethics.

Details of the individual volumes and changes made in subsequent editions are presented in the next section.

### Specific content

In recent years “The biomedical engineering handbook” has been constantly expanded and extended to include further areas of medicine and engineering. The first edition was published in 1995 and won the award: “Winner of the Association of American Publishers Best New Professional/Scholarly Publication—Engineering”. The second edition was published in 2000 by CRC PRESS LLC, Springer in cooperation with IEEE Press. The 2006 edition was also published by CRC Press. The fourth, reviewed edition has been available for readers since 2015, both online and in printed version.

The most obvious difference among these subsequent editions has been length. For example, the second edition has 3024 pages in two volumes, the third edition has 4232 pages (3800 b/w illustrations) in three volumes and the fourth edition has 5430 pages (2060 b/w illustrations) in four volumes. On average, therefore, each subsequent edition is published every 5 or 6 years and extended for additional volumes.

According to the information given by editors in the preface to this latest edition: “*More specifically, this fourth edition has been considerably updated and contains completely new sections, including.*

- *Stem cell engineering*
- *Drug design, delivery systems, and devices*
- Personalized medicine

As well as a number of substantially updated sections, including

- Tissue engineering (which has been completely restructured)
- Transport phenomena and biomimetic systems
- Artificial organs
- Medical imaging
- Infrared imaging
- *Medical informatics*”

In fact, these sections were added and the others expanded with new articles written by new authors. In the preface to the fourth edition, the editors talk about the evolution of the modern healthcare system and then define the term “Biomedical engineering”. At the end of the preface, they distinguish several major divisions of biomedical engineering activities. This division may be controversial for the readers, which I mention later in this review describing the weaknesses of the book. The preface lists the many (570) contributors who were responsible for individual sections of this handbook. I looked up the citation counts of these contributors. In the aggregate, these 153 (43-Vol. I, 14-Vol. II, 30-Vol. III, 66-Vol. IV) authors have themselves published 100 journal articles that have attracted an aggregate number of 40,000 citations. In short, these authors are themselves productive investigators. The most prolific of the authors were David L. Kaplan, Antonios G. Mikos and Jong-Ryoul Kim. It should also be noted that the main editors (Joseph D. Bronzino and Donald R. Peterson) are very prolific authors of many articles and respected researchers in this field.

The thematic scope of the articles is so wide that in this short review it is difficult to mention in detail even the key issues. Individual sections have a number of articles that range from a few to several dozen. The subjects of articles are arranged in order from history through a review of achievements in the considered biomedical engineering area and ending with specialist articles contemplating and solving a specific research problem.

*The first volume* is devoted to the fundamentals of biomedical engineering. It contains basic information in the field of biomechanics, biomaterials, and neuroengineering. Most of the book is richly illustrated both in terms of the presented biological material as well as the schematic and block diagrams of the performed experiments. The articles in the first chapter, namely Physiologic Systems, are extremely important didactically. They provide detailed descriptions of the nervous as well as vision and hearing systems. These are the following articles:

1. Evangelia Micheli-Tzanakou: “Nervous system”, comprising the following, more vital, sections: definitions, functions of the nervous system, representation of information in the nervous system, lateral inhibition, higher functions of the nervous system, abnormalities of the nervous system,
2. Aaron P. Batista and George D. Stetten: “Vision system”, comprising the following, more vital, sections: fundamentals of vision research, a modular view of the vision system, eye movements,
3. Ben M. Clopton and Herbert F. Voigt: “Auditory system”, comprising the following, more vital, sections: overview, peripheral auditory system, central auditory system, pathologies.

The other chapters present a theoretical, simulation and experimental approach. The electronic version of the book enables the reader to see the selected images in higher resolution with a click of the mouse. However, apart from a few cases, no additional materials such as videos or multimedia presentations are included. These could enrich the educational value of the handbook, which is a potential use that the authors mention.

*The second volume* is devoted to biomedical devices and human engineering. Michael R. Neuman, a very senior bioengineer, introduces the volume. He separates the field of biomedical devices into sensors, signal processors as well as display and storage devices. The subsequent articles describe various types of sensors ranging from non-electrical value sensors to electrical values sensors. The next chapter presents biomedical devices ranging from measuring amplifiers through respirators and lasers to optical monitoring. The subsequent chapters are devoted to rehabilitation and clinical engineering. They contain articles in the field of orthopaedics, ophthalmology and others. As in the first volume of the reviewed book, all the articles are very well developed in substance, logically organized, and well edited. The deserving special mention are: “Mechanical ventilation” by Khosrow Behbehani, and “Essentials of anaesthesia” by A. William Paulsen. The second volume ends with the article entitled “Applications of virtual” by Eric Rosow and Joseph Adam.

*The third volume* is devoted to biosignals and biomedical imaging. The four chapters in this volume focus on signal and biomedical image processing, infrared radiation and computer science in biomedical engineering. These chapters contain numerous articles that are at different levels of detail. For example the chapter on signal processing includes articles on spectral analysis and data acquisition. The chapter on biomedical imaging contains articles devoted to imaging in a variety of biomedical devices. The last article entitled “Medical image search” by Thomas Deserno presents an overview of medical image repositories. The most extensive chapter is on infrared imaging. The most important articles include: “Quantitative active dynamic thermal IR-imaging and thermal tomography in medical diagnostics” by Antoni Nowakowski and others. These articles focus on dynamic thermography and technical limitations of its use. The last chapter describes software used in medicine and e-health services, both describing the software and its basic capabilities and scope.

*The fourth volume has 93 articles* devoted to molecular, cellular and tissue engineering. In his brief introduction, Michael M. Domach outlines the scope and organization of the individual chapters. “*Chapter 1 provides some historical background and basic working knowledge…. Chapter 2 is a new contribution that provides an up-to-date look at interactions and the means used to explore and quantify their nature…. Chapter 3 is a new contribution that covers DNA isolation and sequencing. Chapters 4 and 5 present applications in microbial and animal cell systems. Additions in cultivation techniques and long pathway engineering can be found in Chapter 4. RNA silencing methods and examples have been added to update Chapter 5. Chapter 6 is a new contribution that illustrates one medical impact of molecular biology*.” The first article, by Nathan R. Domagalski and Michael M. Domach, entitled “Historical perspective and basics of molecular biology” describes the history of molecular biology showing the individual achievements of scientists in recent years. Other interesting articles include “Expression in mammalian cells” and “Tissue engineering bioreactors”.

### Strengths of the book

This handbook is the largest (for comparison see [1–6]) and most recent summaries of progress in biomedical engineering that currently exists, and it spans the full range of this discipline: fundamentals, biomaterials [7], signals and biomedical images [8, 9] and molecular biology. Some of the authors are the most cited and prolific investigators in the field. All of the articles are at a high level in terms of content, logically arranged, and well edited. These articles are often mini-reviews that provide overview of selected topics. They are generally summaries of material but not critical reviews of research topics. This massive undertaking shows the strong commitment by the editors and publisher (CRC Press) to a successful project, which this certainly is. The handbook will be valuable for educating students in biomedical engineering, and also for physicians and other engineers who come into contact with medicine for their future work. It provides a valuable history of engineering in biology and medicine, and an overview of new advances in the field. Compared to earlier editions, the new material on ethics, medical imaging and molecular biology are particularly welcome. Apart from the hardback volumes (which are sold individually), the availability online will expand its reach. Individual articles can be purchased individually (for $20 per article).

### Weaknesses of the book

Three aspects require some discussion: the purpose, proportion between the chapters and selection of authors and topics.

Purpose. In the preface the authors state that “*It can serve as an excellent textbook for students in areas where traditional textbooks have not yet been developed and as an excellent review of the major areas of activity in each biomedical engineering sub-discipline, such as biomechanics, biomaterials, bioinstrumentation, medical imaging, and so on*.”. Certainly this handbook is far too much to ask a student to buy, and at a cost of 254GBP ($400) would be the reach of most students in any event. It is also too expensive for many faculty or biomedical engineers themselves; in much of the European Union it costs the equivalent of about half of the minimum monthly salary. However, many libraries, even in less affluent countries, purchase online access to this and other such handbooks. For educational purposes the handbook could be a valuable resource and instructors might consider using individual articles to supplement their own courses. For institutions that have not purchased online access, the question arises how to legally distribute this material to students. The lack of the usual didactic material found in textbooks (end of chapter problems, worked examples, callouts with clarifications, online videos and other material) also limit the usefulness of the handbook as a (primary) textbook. On the other hand, the level of material in most chapters is comparable to that taught in most graduate level courses in the field and should be accessible to advanced students.

Proportion my second comment concerns what often seems to be a lack of balance in the volume compared to the real world of medicine and also to biomedical engineering as it is currently practiced. For example, the third volume contains an entire section, with 33 chapters on infrared imaging. This does not match hierarchically the other chapters on signal and image processing—the previous sections in this volume are on biomedical sensors and medical imaging, far broader topics. Secondly, on might ask what is so special about infrared imaging to justify 33 chapters on the topic.

Why is infrared imaging more important than magnetic resonance imaging (MRI, four chapters) or ultrasound or mammography (one chapter each)? MRI is one of the most important imaging technologies to have ever been developed. It is used with countless patients on a routine basis, and is also a fundamental tool in many research programs. Infrared imaging is still a niche field with limited application in medical practice [10].

I assume that the disproportionate attention to infrared imaging reflects the interests of the authors who agreed to write chapters, and the enthusiasm of the section editor. However, the relative attention to different topics in this handbook may not be a reliable guide to the importance of particular topics in present medicine.

As might be expected, the individual chapters vary in levels of detail and approach. An example is the chapter on analysis and image processing in volume III (Biomedical signals, imaging and informatics).

In one group of articles in this section binarization and its consequences are discussed in detail. In other articles, it is ignored and marginalized because of its simplicity. The same basic information is duplicated in some articles. This begs the question to what extent, if at all, the editors worked with the authors to plan the detailed contents of their articles. Given the massive scale of the handbook, it may simply not have been practical to coordinate contents very precisely.

Author and topic selection I looked at the citation records of the authors, and the countries in which they worked. A few of the authors are very highly cited, others had much lower visible impact in the field as judged by citation analysis. That has little directly to do with the quality of the chapters. However, a low citation count may mean that the author is working in a very small subspecialty and his/her chapter, however very well prepared, might not reflect a major effort within the field. That appears to be the case with several chapters on infrared imaging of the breast, for example. In my opinion, the editors should pay more attention to providing a balance of topics in the next edition that more accurately reflects the importance of subfields to medicine at large. One way to do this is to recruit well-known authors with high citation counts to write individual chapters.

Lack of supplementary material. It would be extremely valuable to enrich the articles with supplementary online materials such as videos, presentations etc. These would enhance the usefulness of the book for education, and also allow ordinary readers to gain a better and easier understanding of the material.

These comments should be taken as recommendations and suggestions for future editions. They should not be read as contradicting my overall very good opinion about this handbook.

## Conclusion

This handbook is one of the most interesting and most complete and largest books in the field of biomedical engineering. Apart from the basic information in the field of biomechanics and biomedical devices for signal and biomedical image processing, it also covers the area of molecular biology. It will be a valuable resource for advanced undergraduate students and doctoral students as well as engineers and physicians during specialization. It is continually updated, bringing together articles written by the most famous scientists in the world. Certainly, this book should be available, either in hardcopy in libraries or online, to every student or professional involved in biomedical engineering. Whether an individual scientist should purchase a personal copy of this pricey handbook is a different matter.
